# (*tert*-But­yl)(2-hy­droxy­eth­yl)ammonium chloride

**DOI:** 10.1107/S1600536814012847

**Published:** 2014-06-18

**Authors:** Cintya Valerio-Cárdenas, Simón Hernández-Ortega, David Morales-Morales

**Affiliations:** a Instituto de Química, Universidad Nacional Autónoma de México, Circuito Exterior, Ciudad Universitaria, Coyoacán, México, DF 04510, Mexico

## Abstract

In the cation of the title mol­ecular salt, C_6_H_16_NO^+^·Cl^−^, the N—C—C—O torsion angle is 176.5 (2)°. In the crystal, the cations and chloride ions are linked by N—H⋯O and O—H⋯O hydrogen bonds, generating a two-dimensional network parallel to (100).

## Related literature   

For the chiral pool synthesis of naturally occurring mol­ecules, see: Coppola & Schuster (1987 ▶); Bergmeier & Stanchina (1999 ▶). For pharmacologic synthesis, see: Gante (1994 ▶); Tok & Rando (1998 ▶). 
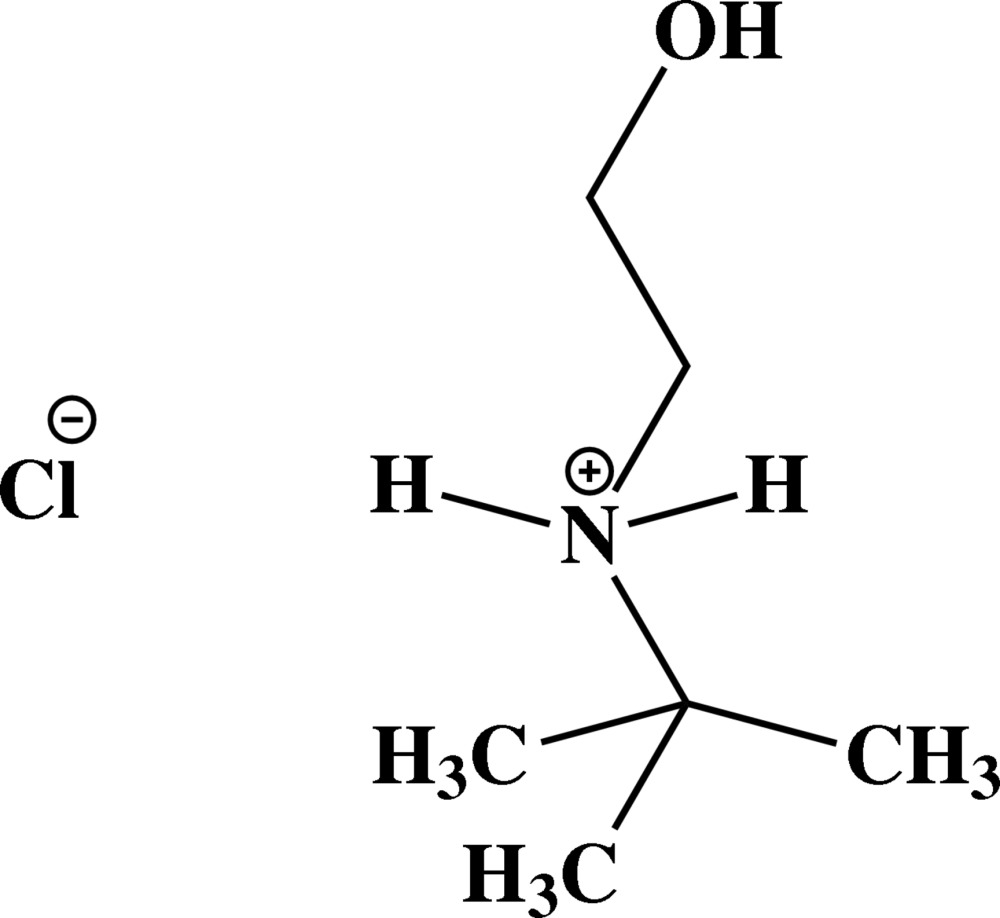


## Experimental   

### 

#### Crystal data  

C_6_H_16_NO^+^·Cl^−^
*M* *_r_* = 153.65
Monoclinic, 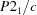

*a* = 8.5204 (3) Å
*b* = 7.8742 (3) Å
*c* = 14.1844 (5) Å
β = 105.804 (1)°
*V* = 915.68 (6) Å^3^
*Z* = 4
Mo *K*α radiation
μ = 0.35 mm^−1^
*T* = 298 K
0.40 × 0.10 × 0.03 mm

#### Data collection  

Bruker APEXII CCD area-detector diffractometer
5487 measured reflections
1668 independent reflections
1071 reflections with *I* > 2σ(*I*)
*R*_int_ = 0.058

#### Refinement  

*R*[*F* ^2^ > 2σ(*F* ^2^)] = 0.046
*wR*(*F* ^2^) = 0.124
*S* = 1.00
1668 reflections
94 parameters
3 restraints
H atoms treated by a mixture of independent and constrained refinement
Δρ_max_ = 0.48 e Å^−3^
Δρ_min_ = −0.25 e Å^−3^

### 

Data collection: *APEX2* (Bruker, 2007 ▶); cell refinement: *SAINT* (Bruker, 2007 ▶); data reduction: *SAINT*; program(s) used to solve structure: *SHELXTL* (Sheldrick, 2008 ▶); program(s) used to refine structure: *SHELXL97* (Sheldrick, 2008 ▶); molecular graphics: *SHELXTL*; software used to prepare material for publication: *SHELXTL*.

## Supplementary Material

Crystal structure: contains datablock(s) I. DOI: 10.1107/S1600536814012847/gw2144sup1.cif

Structure factors: contains datablock(s) I. DOI: 10.1107/S1600536814012847/gw2144Isup2.hkl

Supporting information file. DOI: 10.1107/S1600536814012847/gw2144Isup3.cml

CCDC reference: 1006385

Additional supporting information:  crystallographic information; 3D view; checkCIF report

## Figures and Tables

**Table 1:** Hydrogen-bond geometry (Å, °)

*D*—H⋯*A*	*D*—H	H⋯*A*	*D*⋯*A*	*D*—H⋯*A*
O1—H1⋯Cl1^i^	0.86 (1)	2.29 (1)	3.140 (2)	167 (3)
N3—H3*A*⋯Cl1	0.90 (1)	2.27 (1)	3.144 (2)	166 (2)
N3—H3*B*⋯Cl1^ii^	0.89 (1)	2.30 (1)	3.190 (2)	175 (2)

Symmetry codes: (i) 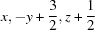; (ii) 
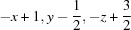
.

**Fig. 1.**
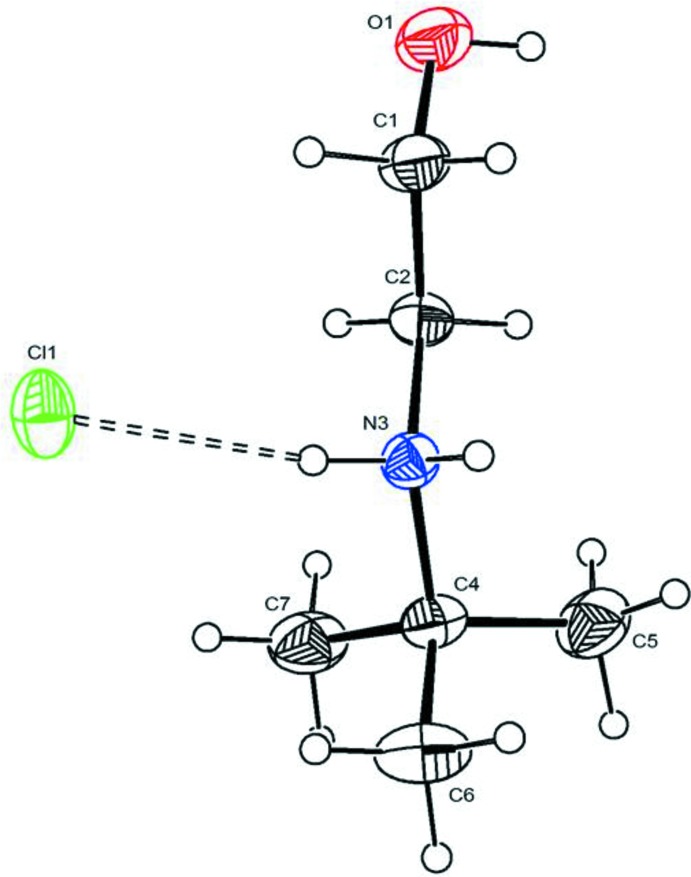
The molecular structure of the title compound showing the atom labelling and displacement ellipsoids at the 40% of probability.

**Fig. 2.**
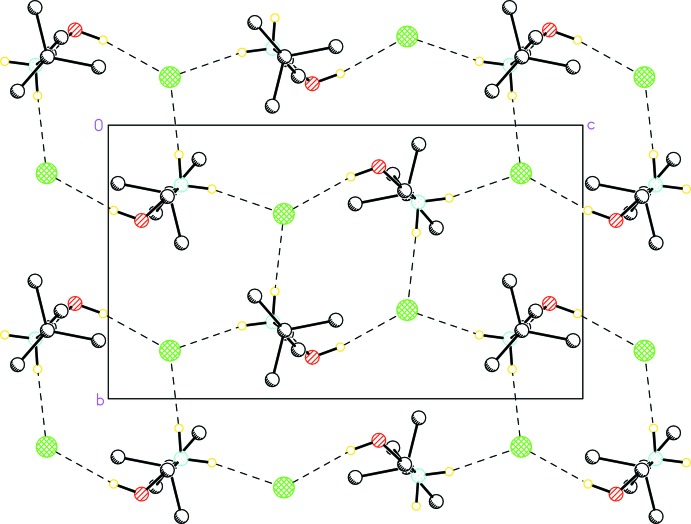
A view in projection on the direction of the chain. The O—H···Cl and N—H···Cl interactions are shown as dashed lines.

## supplementary crystallographic information

### S1. Comment

Amino alcohols are some of the most versatile starting materials both at the laboratory and at the industrial scale and have been widely used for a large number of applications. Among which stands the chiral pool synthesis of naturally occurring molecules (Coppola *et al.* 1987; Bergmeier *et al.* 1999). These compounds have also displayed important biological activities and are of interest for the development of synthetic methods in the pharmaceutical industry (Gante, 1994; Tok *et al.* 1998). Based on the above, we report here the crystal structure of *N*-((2-hydroxyethyl)tertbutyl)ammonium chloride and discuss its geometry and intermolecular interactions.

The molecular structure of the title compound [(HOC_2_H_4_)((CH_3_)_3_C)NH_2_]Cl^-^ (Fig. 1), consists of an ionic species, exhibiting the nitrogen atom in a tetrahedral geometry. The dihedral angle between the tertbutyl and the 2-hydroxyethyl moieties is almost plane (173.34 (2)°) as a result of the reduced steric effects. In the asymmetric unit the Cl atom is linked by a N3—H3A···Cl1 interaction (2.266 (11) Å). In the crystal the Cl atom is acting as tri-acceptor H-bonding, such that, the anion and cation species are linked through O1—H1···Cl1 with distances of 2.294 (13) Å, leading to stairs aligned along the *ac* plane (symmetry code *x*, -*y* + 3/2, *z* + 1/2). These stairs are expanded by a third intermolecular interaction N3—H3B···Cl1 (2.304 (10) Å) along the *b* axis with symmetry code -*x* + 1,*y* - 1/2,-*z* + 3/2 (see Table 1, Fig. 2).

### S2. Experimental

The title compound was isolated from the reaction of [S_2_CN(*^t^*Bu)(EtOH)] and [NiCl_2_(PPh_3_)_2_] in a 1:1 molar ratio in ethanol. Colourless crystals suitable for single-crystal X-ray diffraction analysis were obtained from a solvent system ether/CH_2_Cl_2_.

### S3. Refinement

The atoms H1, H3A and H3B were located from a difference Fourier map and N3—H3A, N3—H3B and O1—H1 distances are restrained to 0.90 and 0.85 Å respectively. H atoms were included in calculated position (C—H = 0.97 Å for methylene H, and C—H = 0.96 Å for methyl H), and refined using a riding model with *U*_iso_(H) = 1.2 *U*_eq_ of the carrier atoms. 3 badly fitting reflections were omitted from the final refinement.

### Crystal data

**Table:**

C_6_H_16_NO^+^·Cl^−^	*F*(000) = 336
*M**_r_* = 153.65	*D*_x_ = 1.115 Mg m^−^^3^
Monoclinic, *P*2_1_/*c*	Mo *K*α radiation, λ = 0.71073 Å
Hall symbol: -P 2ybc	Cell parameters from 2475 reflections
*a* = 8.5204 (3) Å	θ = 2.5–25.3°
*b* = 7.8742 (3) Å	µ = 0.35 mm^−^^1^
*c* = 14.1844 (5) Å	*T* = 298 K
β = 105.804 (1)°	Prism, colourless
*V* = 915.68 (6) Å^3^	0.40 × 0.10 × 0.03 mm
*Z* = 4	

### Data collection

**Table:**

Bruker APEXII CCD area-detector diffractometer	*R*_int_ = 0.058
Detector resolution: 0.83 pixels mm^-1^	θ_max_ = 25.3°, θ_min_ = 2.5°
ω scans	*h* = −5→10
5487 measured reflections	*k* = −8→9
1668 independent reflections	*l* = −17→16
1071 reflections with *I* > 2σ(*I*)	

### Refinement

**Table:**

Refinement on *F*^2^	3 restraints
Least-squares matrix: full	Hydrogen site location: inferred from neighbouring sites
*R*[*F*^2^ > 2σ(*F*^2^)] = 0.046	H atoms treated by a mixture of independent and constrained refinement
*wR*(*F*^2^) = 0.124	*w* = 1/[σ^2^(*F*_o_^2^) + (0.0584*P*)^2^] where *P* = (*F*_o_^2^ + 2*F*_c_^2^)/3
*S* = 1.00	(Δ/σ)_max_ = 0.001
1668 reflections	Δρ_max_ = 0.48 e Å^−^^3^
94 parameters	Δρ_min_ = −0.25 e Å^−^^3^

### Special details

**Table:**

Geometry. All e.s.d.'s (except the e.s.d. in the dihedral angle between two l.s. planes) are estimated using the full covariance matrix. The cell e.s.d.'s are taken into account individually in the estimation of e.s.d.'s in distances, angles and torsion angles; correlations between e.s.d.'s in cell parameters are only used when they are defined by crystal symmetry. An approximate (isotropic) treatment of cell e.s.d.'s is used for estimating e.s.d.'s involving l.s. planes.

### Fractional atomic coordinates and isotropic or equivalent isotropic displacement parameters (Å^2^)

**Table:**

	*x*	*y*	*z*	*U*_iso_*/*U*_eq_	
Cl1	0.44289 (9)	0.82425 (9)	0.62991 (4)	0.0579 (3)	
O1	0.2643 (2)	0.8483 (3)	0.92991 (15)	0.0690 (6)	
H1	0.303 (4)	0.811 (4)	0.9889 (11)	0.083*	
C1	0.3512 (3)	0.7606 (4)	0.87548 (19)	0.0531 (7)	
H1A	0.3003	0.7781	0.8062	0.064*	
H1B	0.3488	0.6400	0.8889	0.064*	
C2	0.5253 (3)	0.8207 (3)	0.90041 (18)	0.0436 (6)	
H2A	0.5280	0.9397	0.8832	0.052*	
H2B	0.5742	0.8098	0.9704	0.052*	
N3	0.6212 (2)	0.7191 (3)	0.84643 (15)	0.0383 (5)	
H3A	0.579 (3)	0.734 (3)	0.7818 (8)	0.046*	
H3B	0.607 (3)	0.6079 (13)	0.8511 (17)	0.046*	
C4	0.8038 (3)	0.7496 (3)	0.87151 (19)	0.0454 (7)	
C5	0.8775 (3)	0.7126 (4)	0.9796 (2)	0.0686 (9)	
H5A	0.8423	0.6028	0.9950	0.082*	
H5B	0.8427	0.7976	1.0181	0.082*	
H5C	0.9944	0.7142	0.9941	0.082*	
C6	0.8687 (3)	0.6245 (4)	0.8087 (2)	0.0728 (9)	
H6A	0.8452	0.5105	0.8247	0.087*	
H6B	0.9845	0.6386	0.8214	0.087*	
H6C	0.8172	0.6459	0.7407	0.087*	
C7	0.8344 (3)	0.9303 (4)	0.8456 (2)	0.0678 (9)	
H7A	0.9497	0.9489	0.8584	0.081*	
H7B	0.7906	1.0071	0.8844	0.081*	
H7C	0.7825	0.9498	0.7774	0.081*	

### Atomic displacement parameters (Å^2^)

**Table:**

	*U*^11^	*U*^22^	*U*^33^	*U*^12^	*U*^13^	*U*^23^
Cl1	0.0865 (6)	0.0441 (4)	0.0413 (4)	0.0031 (3)	0.0142 (4)	−0.0011 (3)
O1	0.0533 (13)	0.0912 (16)	0.0646 (13)	0.0179 (11)	0.0194 (11)	0.0001 (13)
C1	0.0387 (17)	0.0772 (19)	0.0429 (15)	0.0068 (14)	0.0103 (12)	−0.0066 (15)
C2	0.0407 (16)	0.0462 (15)	0.0467 (14)	0.0013 (11)	0.0166 (12)	−0.0050 (13)
N3	0.0377 (13)	0.0364 (11)	0.0406 (11)	0.0007 (9)	0.0101 (10)	−0.0003 (11)
C4	0.0342 (15)	0.0438 (14)	0.0584 (17)	0.0013 (11)	0.0130 (13)	−0.0028 (14)
C5	0.0472 (19)	0.084 (2)	0.0657 (19)	0.0039 (15)	−0.0004 (15)	0.0017 (18)
C6	0.0513 (19)	0.079 (2)	0.095 (2)	0.0051 (16)	0.0311 (17)	−0.020 (2)
C7	0.0463 (18)	0.0607 (19)	0.099 (2)	−0.0077 (14)	0.0232 (17)	0.0038 (19)

### Geometric parameters (Å, º)

**Table:**

O1—C1	1.390 (3)	C4—C5	1.519 (4)
O1—H1	0.862 (10)	C4—C6	1.529 (4)
C1—C2	1.504 (4)	C5—H5A	0.9600
C1—H1A	0.9700	C5—H5B	0.9600
C1—H1B	0.9700	C5—H5C	0.9600
C2—N3	1.495 (3)	C6—H6A	0.9600
C2—H2A	0.9700	C6—H6B	0.9600
C2—H2B	0.9700	C6—H6C	0.9600
N3—C4	1.518 (3)	C7—H7A	0.9600
N3—H3A	0.896 (9)	C7—H7B	0.9600
N3—H3B	0.888 (10)	C7—H7C	0.9600
C4—C7	1.510 (4)		
			
C1—O1—H1	104 (2)	C7—C4—C6	110.5 (2)
O1—C1—C2	110.7 (2)	N3—C4—C6	105.8 (2)
O1—C1—H1A	109.5	C5—C4—C6	110.4 (2)
C2—C1—H1A	109.5	C4—C5—H5A	109.5
O1—C1—H1B	109.5	C4—C5—H5B	109.5
C2—C1—H1B	109.5	H5A—C5—H5B	109.5
H1A—C1—H1B	108.1	C4—C5—H5C	109.5
N3—C2—C1	110.6 (2)	H5A—C5—H5C	109.5
N3—C2—H2A	109.5	H5B—C5—H5C	109.5
C1—C2—H2A	109.5	C4—C6—H6A	109.5
N3—C2—H2B	109.5	C4—C6—H6B	109.5
C1—C2—H2B	109.5	H6A—C6—H6B	109.5
H2A—C2—H2B	108.1	C4—C6—H6C	109.5
C2—N3—C4	117.61 (19)	H6A—C6—H6C	109.5
C2—N3—H3A	109.4 (16)	H6B—C6—H6C	109.5
C4—N3—H3A	108.7 (16)	C4—C7—H7A	109.5
C2—N3—H3B	112.9 (15)	C4—C7—H7B	109.5
C4—N3—H3B	106.6 (15)	H7A—C7—H7B	109.5
H3A—N3—H3B	100 (2)	C4—C7—H7C	109.5
C7—C4—N3	109.1 (2)	H7A—C7—H7C	109.5
C7—C4—C5	112.0 (2)	H7B—C7—H7C	109.5
N3—C4—C5	108.8 (2)		

### Hydrogen-bond geometry (Å, º)

**Table:**

*D*—H···*A*	*D*—H	H···*A*	*D*···*A*	*D*—H···*A*
O1—H1···Cl1^i^	0.86 (1)	2.29 (1)	3.140 (2)	167 (3)
N3—H3*A*···Cl1	0.90 (1)	2.27 (1)	3.144 (2)	166 (2)
N3—H3*B*···Cl1^ii^	0.89 (1)	2.30 (1)	3.190 (2)	175 (2)

Symmetry codes: (i) *x*, −*y*+3/2, *z*+1/2; (ii) −*x*+1, *y*−1/2, −*z*+3/2.
